# Systematic gene-disease relationship (GDR) curation unveils 61 gene-disease associations and highlights the impact on genetic testing

**DOI:** 10.1016/j.gimo.2023.100833

**Published:** 2023-09-09

**Authors:** Emir Zonic, Mariana Ferreira, Luba M. Pardo, Javier Martini, Maria Eugenia Rocha, Ruxandra Aanicai, Natalia Ordonez-Herrera, Deepa Saravanakumar, Ligia S. Almeida, Inês C. Fernandes, Nishtha Gulati, Sumanth Mannepalli, Amela Hercegovac, Ruslan Al-Ali, Catarina Pereira, Omid Paknia, Uros Hladnik, Peter Bauer, Jorge Pinto Basto, Aida M. Bertoli-Avella

**Affiliations:** 1 CENTOGENE GmbH, Rostock, Germany; 2 University of Tuzla, Faculty of Natural Sciences and Mathematics, Bosnia and Herzegovina; 3 University of Rostock, Rostock, Germany

**Keywords:** Gene classification, Gene curation, Gene-disease association, Gene-disease relationship, Gene-validity

## Abstract

**Purpose:**

With this study, we aimed to explore the gene-disease relationship (GDR) evidence for 109 gene-disease pairs and the significance of a large Biodatabank for this classification.

**Methods:**

The Clinical Genome Resource (ClinGen) Clinical Validity Framework for evaluation of GDR was applied. Most of the assessed genes were without a phenotype entry in the Online Mendelian Inheritance in Man (OMIM) database. Our Biodatabank with genetic data from over 670,000 previously tested individuals, in addition to data available in literature and public databases, were used for gene curation.

**Results:**

We confirmed 61 GDR (Definitive: 4 genes, Strong: 22 genes, Moderate: 35 genes). For 84 of 109 gene-disease pairs, a higher score was reached when using data from our Biodatabank in addition to externally obtainable data. This increased the final level of classification in 21 of the genes. Over 400 patients received a genetic report with clinically relevant variants in these 61 genes.

**Conclusion:**

Our results demonstrate the importance of careful assessment of gene clinical validity data, along with the use of genetic data repositories. Implementation of the ClinGen Clinical Validity Framework for assessment of GDR is relatively straightforward. We encourage diagnostic laboratories to implement such a system and contribute to closing the knowledge gap in genetic research and diagnostics.

## Introduction

In the last years, with the widespread application of exome and genome analysis, an increased number of variants in newly discovered genes has been reported.^1^ However, many genes still have a limited or uncertain diagnostic value, with the translation of the genomic research findings into the clinical setting taking years. Many patients remain undiagnosed even after using exome/genome sequencing.^2^, ^3^, ^4^ Historically, the description of single patients was often the starting point for the identification of novel monogenic diseases.^5^ Although this could be sufficient for diseases with low clinical and genetic heterogeneity, for many disorders, the availability of multiplex or consanguineous families was an important contribution for these advances, making use of different approaches, such as linkage analyses and experimental/functional data. The recognition of additional patients with the same condition was then easier, allowing for a new clinical entity to be defined and an inheritance mode to be suggested.^6^^,^^7^ More recently, large-scale sequencing data have enabled different approaches to gene discovery via the identification of rare and gene-disrupting variants in larger subsets of patients, when compared with population controls.^6^

Evaluating the clinical impact of genetic variants is a challenge, with the first step being the establishment of gene-disease relationship (GDR). There is considerable variability in the level of evidence supporting a causal relationship between genotype and phenotype; in many instances, a formal and systematic evaluation is needed to differentiate clinically valid relationships from less well-substantiated associations. The National Institutes of Health (NIH)-funded ClinGen group developed a framework to systematically ascertain the level of evidence of gene-phenotype relationships using qualitative and quantitative assessments. The framework evaluates genetic and experimental evidence to classify GDR into 6 classes of strength of evidence ranging from Definitive evidence to contradictory evidence. ClinGen also uses a semi-quantitative system that weights the genetic and experimental evidence using a score system that ranges from 0 for no evidence to a maximum of 18 for a high level of genetic and experimental evidence, with a score exceeding 12 indicating Strong or Definitive evidence.^8^^,^^9^ The classification derived by the framework is reviewed by experts in the field and may be modified based on their expert opinion. Furthermore, researchers can review and download information of specific genes in a public repository. A detailed description for using the framework and access to the curation interface is available.^8^^,^^9^

The validity of a GDR is of utmost importance for an accurate interpretation of the genetic findings and to develop gene panels for routine diagnostic use.^10^ Current guidance suggests that only those gene-disease pairs with a confirmed GDR, ie, with clinical validity of moderate or above, should be included in clinical testing.^10^ The implementation of ClinGen guidelines can help to standardize the annotation of genes to new diseases and should be the starting point and a key step in a diagnostic setting. The Gene Curation Coalition (GenCC) is aiming to harmonize this approach and nomenclature to ensure that gene-level curation is comparable between institutions.^9^

Gene-disease curation can often be challenging because of minimal information being available in the literature and a limited number of patients having been reported. Within this study, we used the ClinGen framework to evaluate its feasibility and application in the day-to-day work of a diagnostic laboratory. We systematically evaluated genes without OMIM entries or with entries indicating that the relationship between the phenotype and gene was provisional. The gene selection depended on the specific variants identified during the diagnostic evaluation of patients’ genomic data. Within this work, we described our experience and results after assessing 107 genes for 109 GDRs.

## Materials and Methods

### The Clinical Genome Resource (ClinGen)

The ClinGen Clinical Validity Framework for the evaluation of GDR was applied. The framework is designed to evaluate relevant genetic and experimental evidence needed to consolidate or exclude the clinical relevance of a gene in Mendelian disorders based on a standardized evidence assessment. A comprehensive explanation of ClinGen is presented elsewhere.^8^^,^^9^ Briefly, the strength of evidence for a GDR can be categorized as Definitive, Strong, Moderate, Limited, or No Known Disease Relationship. The total number of needed points for Definitive and Strong association according to the framework is 12 to 18, for Moderate association 7 to 11, and for Limited gene-validity 1 to 6 points. The main difference between a Definitive and Strong relationship is that, for the former, the role of the gene in the disease needs to be repeatedly demonstrated in both the research and clinical settings and upheld over time (usually at least 2 independent studies over 3 years). In the case of Contradictory evidence, when conflicting data were reported, Disputed and Refuted categories were also an option in the final classification. Evidence taken into consideration according to the ClinGen’s framework comprise genetic and experimental evidence. Under genetic evidence case-level data or proband data, segregation data, and case-control data can be scored with maximum points of 12 for genetic evidence. For experimental evidence, the maximum points are 6, and it has 3 different categories, including function, functional alteration, and animal and rescue models. The functional evidence category includes biochemical, protein interaction, and expression evidence type, whereas functional alteration includes cells from affected individual and engineered cells. In the end, models and rescue models have 4 different types of evidence: animal model, cell culture model system, rescue in animal model, and rescue in engineered equivalent. We used the same criteria as the ClinGen framework for further analyses.

ClinGen has also developed recuration recommendations for the previously assessed GDRs, providing an interval for re-evaluation for all categories (https://clinicalgenome.org/site/assets/files/2164/clingen_standard_gene-disease_validity_recuration_procedures_v1.pdf). In our genetic diagnostic setting, the recuration process occurs when a new case is identified during the diagnostics process or in the internal Biodatabank or when other family members are tested.

The newly described diseases without OMIM entry have been named in this work following the recent ClinGen's disease-naming guidance (https://clinicalgenome.org/docs/clingen-guidance-and-recommendations-for-monogenic-disease-nomenclature/). In short, the gene name and the major phenotypic feature(s) or spectrum, eg, *BRSK2*-related neurodevelopmental disorder or *NRAP*-related dilated cardiomyopathy are used. Two formats can be applied for monogenic disease entity nomenclature, eg, *RYR1*-related malignant hyperthermia or malignant hyperthermia related to *RYR1.*

### Our Biodatabank repository

To assess the feasibility of the ClinGen framework in a diagnostic setting, we used our Biodatabank repository. This database contains data from over 670,000 individuals represented from over 120 highly diverse countries (November 2022). The annotation of genetic data involves the collection, association, update, and review of genetic and phenotypic data in a standardized, structured manner. A description of the repository can be found elsewhere.^11^ It is important to stress that the Biodatabank includes a comprehensive curation step, which helps to maintain the integrity of the data over time. As recommended by ClinGen’s gene-validity framework, a case should only be taken into consideration if sufficient clinical information is available, preferably in the form of Human Phenotype Ontology (HPO) codes and/or text.

### Candidate gene selection

**Figure 1:**
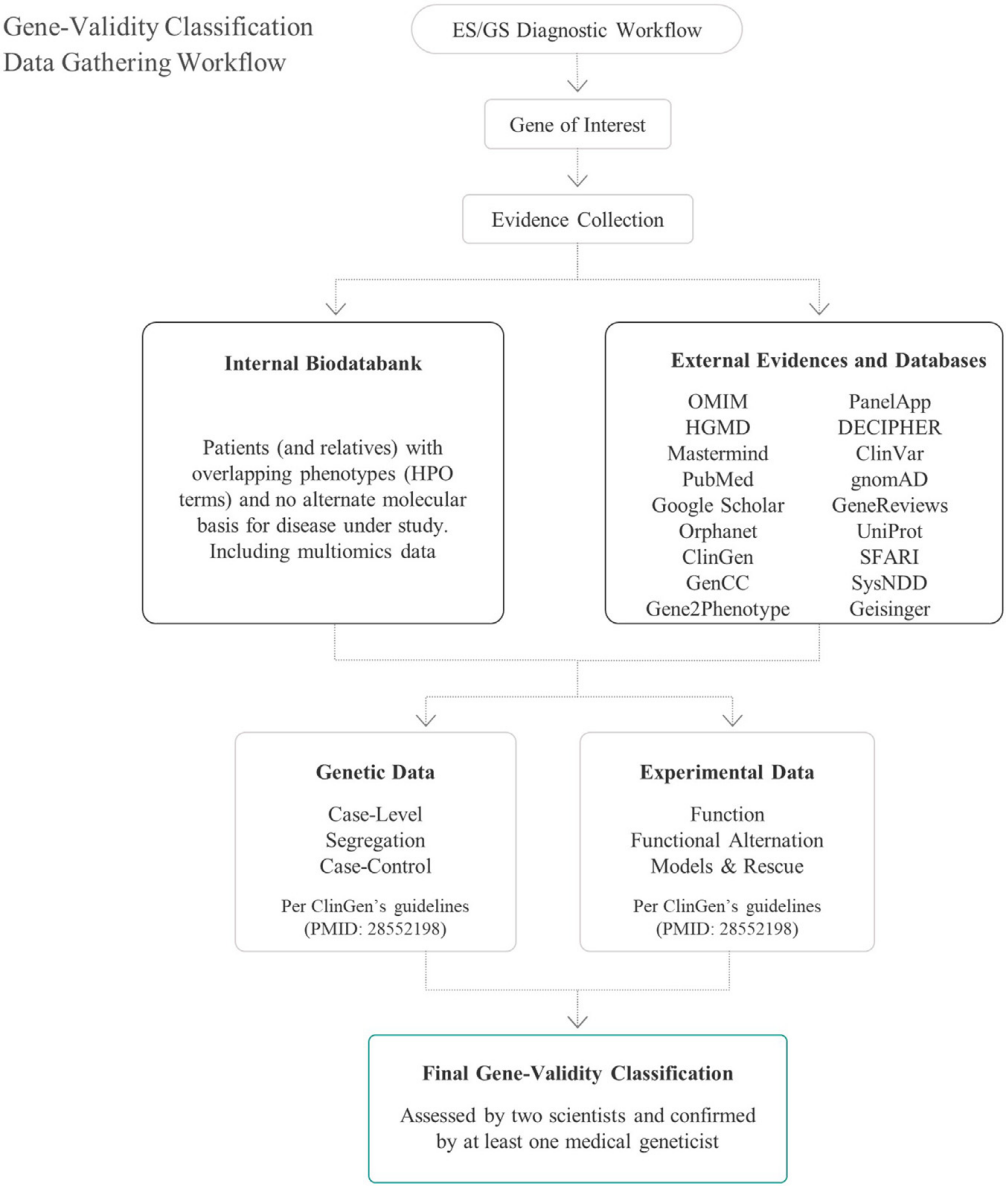
**Workflow followed for gene-disease selection and evaluation.** Genes evaluated from a period of 11 months are included in this work (January 2022-November 2022).

Figure 1 presents the workflow of our study. In total, 107 genes (109 gene-disease pairs as 2 genes were evaluated for both autosomal dominant and autosomal recessive mode of inheritance [MOI]) were selected for evaluation after relevant variants were detected by exome/genome analysis in relation to the patients’ clinical phenotype during the period from January 2022 to November 2022. The inclusion criteria were as follows: (1) limited publicly available gene-disease assessment and/or (2) no phenotypic information for the gene in the OMIM database, or the information in this database was marked as provisional. We also included genes with no information in the GenCC database. Genes with OMIM phenotype entries were mainly assessed for level of association regarding variant classification, especially for assessment of PVS1 criterion used for interpreting loss of function (LoF) variants.^12^ Two curators (E.Z. and M.F.) reviewed the evidence for each gene based on the available literature and our repository. Databases included OMIM, PubMed, ClinGen, GenCC, Gene2Phenotype, PanelApp, ClinVar, DECIPHER, Google Scholar, Mastermind, GeneReviews, UniProt, gnomAD, HGMD, SFARI, SysNDD, and Geisinger (links provided at the end of the manuscript). Genes were classified using the qualitative and the semi-quantitative assessment according to ClinGen. The final gene assessment was done by a medical geneticist (A.M.B.-A. and J.P.B.).

As per the framework guidelines, curating only the amount of information sufficient to reach the maximum number of points was recommended because of the exhaustive literature review typically needed during the classification process. Accordingly, 12 points are required to reach the Strong classification.

Variant nomenclature in the assessed genes followed standard Human Genome Variation Society recommendations. Several aspects were checked for variants under evaluation, such as the type of a variant (ie, null or missense), MOI, disease mechanism (ie, LoF, gain of function, or de novo), phenotype variability, variant frequency in general population, and availability of functional data on variant level (Figure 1).

## Results

**Figure 2:**
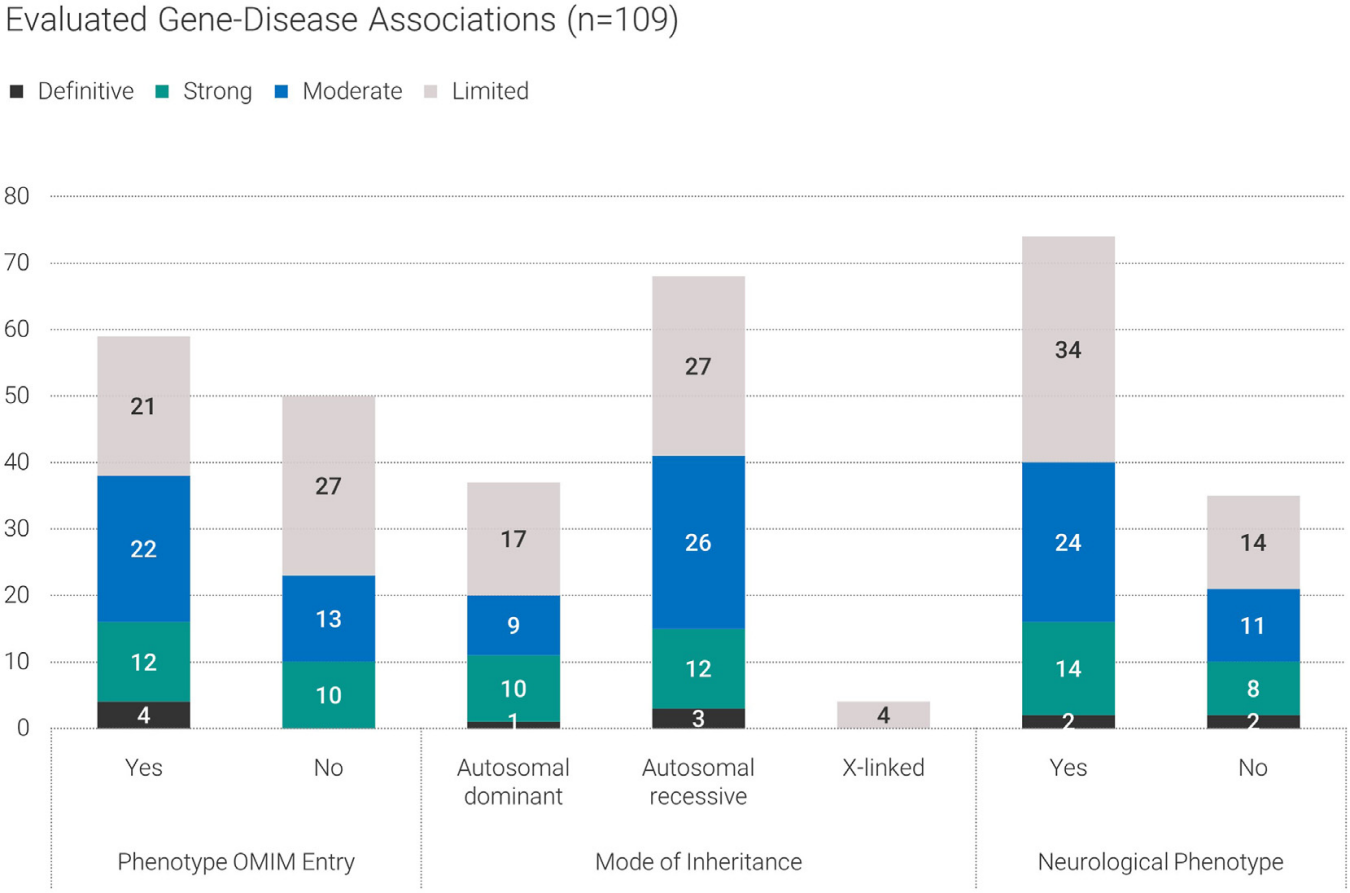
**Summary of the evaluated genes according to pre-existing OMIM phenotype entry, mode of inheritance, andclinical phenotype.**

We evaluated the evidence supporting GDR for 107 genes (109 gene-disease pairs) that were analyzed in our laboratory from January to November 2022 (Figure 1, Supplemental Table 1), using the ClinGen framework. The genes were systematically selected after detecting relevant variants during the evaluation of patient genomic sequence data. As shown in Figure 2, 44 gene-disease pairs (40%) had an associated phenotype in OMIM (3 of these had a different phenotype in our data repository or in other databases or literature), and 15 genes (14%) had only provisional OMIM entry (2 of these were evaluated in the context of a different phenotype, *n* = 59). Of the remaining, 50 gene-disease pairs (46%) did not have an OMIM associated phenotype.

### MOI and phenotypes

Of the 107 evaluated genes, 68 (63%) were associated with autosomal recessive disorders and 37 (34%) with autosomal dominant disorders (Figure 2). X-linked MOI was observed in 4 genes (*SLC25A43, SMARCA1, COL4A6*, and *GYG2*). Two genes (*KDM5A* and *KRS2*) were associated and investigated in the context of both autosomal dominant and recessive inheritance. ClinGen developed lumping and splitting guidelines, an important precuration process, to better define disease entity for use in gene-disease clinical validity evaluations.^13^ The lumping and splitting guidelines stress assertion, molecular mechanism, expressivity, and inheritance pattern as the most important criteria in this process. Curation of the *KDM5A* and *KRS2* genes with both autosomal dominant and autosomal recessive patterns was performed solely based on MOI. Thus, following the guidelines, it is recommended to lump these genes under a single disease spectrum because there is not enough evidence to consider 2 distinct disease entities. Furthermore, the *NOTCH3* gene was evaluated in the context of the autosomal recessive inheritance because several patients in our repository had biallelic *NOTCH3* variants. Previously, this gene was associated with autosomal dominant cerebral arteriopathy with subcortical infarcts and leukoencephalopathy type 1 (CADASIL, OMIM: 125310) and lateral meningocele syndrome (OMIM: 130720) and provisionally associated with infantile myofibromatosis type 2 (OMIM: 615293). In our laboratory, it has been evaluated for autosomal recessive *NOTCH3*-related early-onset leukoencephalopathy.^14^ Based on the lumping and splitting guidelines, there is enough evidence (assertion, MOI, and molecular mechanism) to split this condition under a “new” disease entity, namely, *NOTCH3*-related early-onset leukoencephalopathy.

The majority of the 109 evaluated GDRs were related to a neurological phenotype (*n* = 74, 68%), whereas 35 (32%) were related to variable types of genetic diseases (Figure 2, Supplemental Table 1).

### Levels of evidence

Supplemental Figure 1 presents the number of genes and their distinct levels of evidence reached. The largest proportion of genes were classified as Limited evidence (*n* = 48; 44%), followed by Moderate and Strong level of evidence (*n* = 35; 32% and *n* = 22; 20%, respectively). Also, the median value of the scoring per level of evidence is shown. The total median value for the genes in the category Limited, Moderate, and Strong were 3.5, 8.15, and 13 points, respectively. Experimental data contributed with a modest increase in scores ranging from 0.5 to 4 points, with the largest change observed for *PAPPA2* that increased to 12 points after adding 6 points on experimental evidence. As shown in this figure, genes assigned to Definitive and Strong categories had similar point distribution. The difference between the 2 classes is that for the Definitive level, the role of a gene has been upheld over time, generally for a period of at least 3 years, without conflicting results of the association between the gene-disease pair for the specific disorder. On the other hand, the gene-disease pairs with a Strong classification have been reported more recently.

When looking at all 107 investigated genes (109 gene-disease pairs), the initial score for 84 genes (79%) increased as a result of combining external data with our Biodatabank, with a median increase of 1 point and a range from 0.5 to 10 points (Supplemental Figure 1). Remarkably, including information from Biodatabank changed the level of association for 21 genes, which has a direct impact on the diagnostic yield, because more genes can be considered as diagnostically relevant (Table 1, Figure 2).

**Table 1:** Final classification of 21 genes that changed the level of evidence in this study

Gene	MOI	Phenotype (OMIM Phenotype in Bold)	Genetic Data	Experimental Data	Points Without Own Repository Data	Classification Without Own Repository Data	Total Points	Final Classification
*NOTCH3*	AR	*NOTCH3*-related early-onset leukoencephalopathy	12	2	4.6	Limited	14	Strong
*SCLT1*	AR	*SCLT1*-related multisystem ciliopathy	12	1.5	8.25	Moderate	13.5	Strong
*KCTD3*	AR	*KTCD3*-related developmental delay and seizure	12	0.5	6.5	Limited	12.5	Strong
*FAT1*	AR	*FAT1*-related colobomatous-microphthalmia, ptosis, nephropathy, and syndactyly	9	3.5	10.5	Moderate	12.5	Strong
*NRAP*	AR	*NRAP*-related dilated cardiomyopathy	12	0.5	9.5	Moderate	12.5	Strong
*PAPPA2*	AR	**Dauber-Argente type of short stature**	6.15	6	10.15	Moderate	12.15	Strong
*PPP1R21*	AR	**Neurodevelopmental disorder with hypotonia, facial dysmorphism, and brain abnormalities**	12	0	8.5	Moderate	12	Strong
*C1orf127*	AR	*C1orf127*-related heterotaxia	7.6	2	0	Limited	9.6	Moderate
*ZNF699*	AR	**DEGCAGS syndrome**	8.5	0.5	0.5	Limited	9	Moderate
*FBRSL1*	AD	*FBRSL1*-related malformation and intellectual disability syndrome	6.5	1.5	6	Limited	8	Moderate
*THPO*	AR	*THPO*-related congenital amegakaryocytic thrombocytopenia	4.7	3	5.95	Limited	7.7	Moderate
*RBL2*	AR	**Brunet-Wagner neurodevelopmental syndrome**	6.5	1	6.5	Limited	7.5	Moderate
*CNPY3*	AR	**Developmental and epileptic encephalopathy type 60**	6.5	1	4	Limited	7.5	Moderate
*PAN2*	AR	*PAN2*-related syndromic neurodevelopmental disorder and multiple congenital anomalies	7	0.5	4.5	Limited	7.5	Moderate
*VPS50*	AR	**Neurodevelopmental disorder with microcephaly, seizures, and neonatal cholestasis**	6	1.5	3.5	Limited	7.5	Moderate
*SMG8*	AR	**Alzahrani-Kuwahara syndrome**	6.75	0.5	6.25	Limited	7.25	Moderate
*TMEM218*	AR	**Joubert syndrome type 39**	3.1	4	6.85	Limited	7.1	Moderate
*HID1*	AR	**Developmental and epileptic encephalopathy 105 with hypopituitarism**	6.6	0.5	5.1	Limited	7.1	Moderate
*NME5*	AR	**Primary ciliary dyskinesia type 48, without situs inversus**	3.5	3.5	5.5	Limited	7	Moderate
*AGR2*	AR	**Respiratory infections, recurrent, and failure to thrive with or without diarrhea**	5	2	2	Limited	7	Moderate
*ACBD6*	AR	*ACBD6*-related intellectual disability	7	0	0	Limited	7	Moderate

*MOI*; mode of inheritance; *AR*, autosomal recessive; *AD*, autosomal dominant.

**Figure 3:**
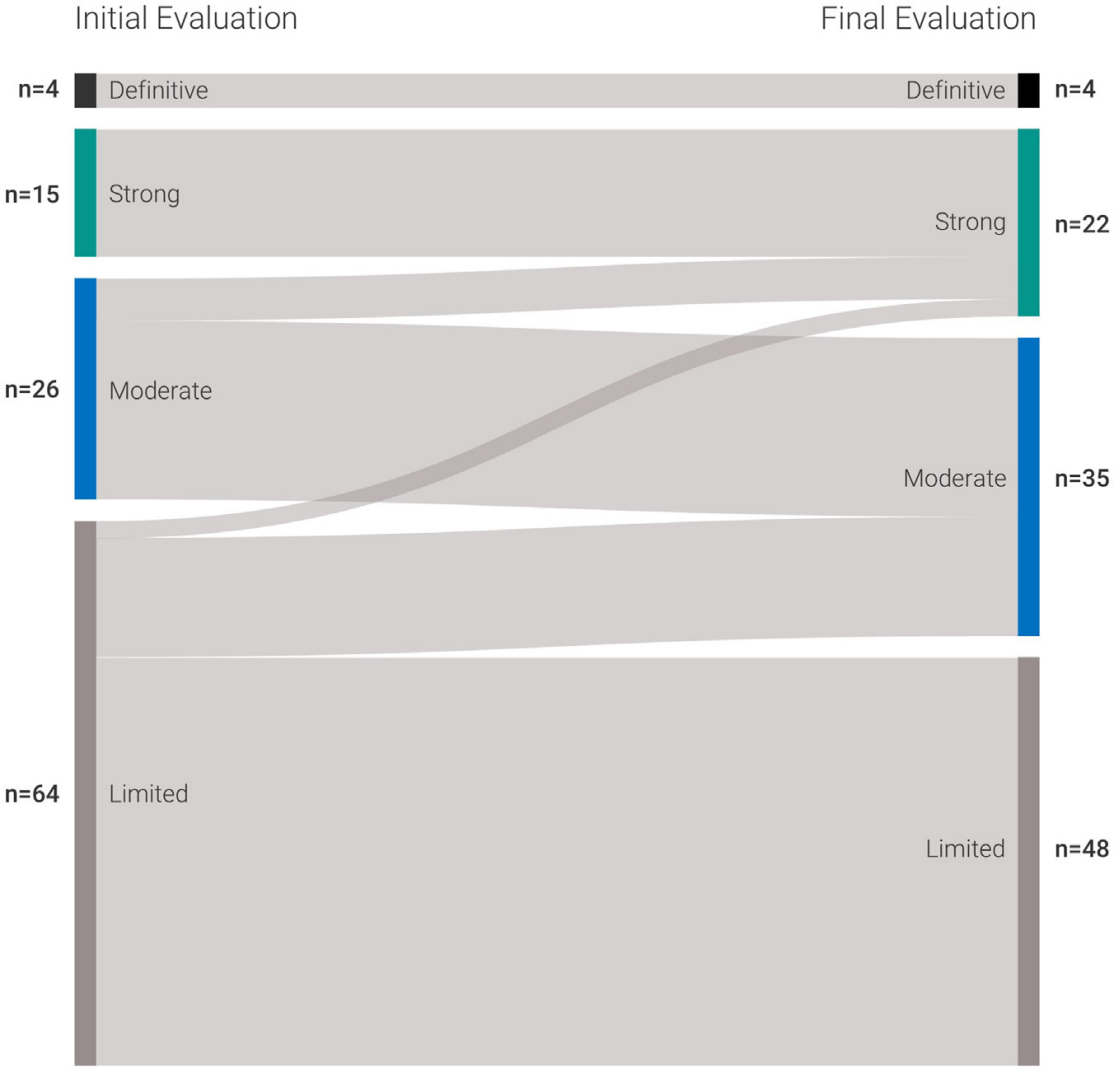
**Sankey diagram illustrating the changes in level of evidence of the 109 GDR evaluated.** Fourteen genes changed from Limited to Moderate; 5 changed from Moderate to Strong; 2 changed from Limited to Strong.

Changes in the evidence category for GDR are summarized in Figure 3. The largest increase in points was seen for 2 genes classified previously as Limited evidence, but that reached 12-point threshold in this analysis, namely, *NOTCH3* (autosomal recessive early-onset leukoencephalopathy) and *KCTD3 (*autosomal recessive neurodevelopmental disorder). Other genes were upgraded from Moderate to Strong, namely, the following: *SCLT1*, *FAT1*, *PPP1R21,* and *NRAP*. In addition, 14 genes with previous Limited evidence were upgraded to genes with Moderate evidence, with 3 genes presenting an increment of 7 points, which means there was little or no information regarding GDR before this study. The remaining genes were classified as Limited evidence and considered as non-diagnostic (Figure 3, Supplemental Table 1).

Interestingly, we observed 1 discrepancy between the OMIM phenotype assigned for the *THSD1* gene and the phenotype of our patients for the same gene. In OMIM, *THSD1* is listed with provisional association with intracranial berry aneurysm type 12, whereas we evaluated this gene in relation to non-immune hydrops fetalis. In GenCC, this gene was listed with Definitive association for autosomal recessive non-immune hydrops fetalis (2021, King Faisal Specialist Hospital and Research Center), Limited for autosomal recessive multiple congenital anomalies/dysmorphic syndrome (2018, Ambry Genetics), and Limited/Strong/Supportive for intracranial berry aneurysm (2018, Ambry Genetics; 2020, Invitae; 2021 Orphanet). A similar scenario was observed for *RAP1GDS1*, *THPO*, *SOX17*, and *ATP8A2.* For example, *RAP1GDS1* is registered in OMIM with a T cell acute lymphocytic leukemia phenotype,^15^ but we analyzed it and published the data in the context of autosomal recessive syndromic intellectual disability.^1^^,^^16^

### Selected examples

#### *KCTD3:* A new GDR - autosomal recessive neurodevelopmental disorder with encephalopathy (Limited to Strong level)

We identified 2 genes that reached the level of Strong association and that had a Limited level of evidence before this study, both without an associated OMIM phenotype. One of these genes was *KCTD3*, evaluated in relation to developmental delay and epilepsy. *KCTD3* encodes for a potassium channel l tetramerization-domain-containing protein. The protein binds a hyperpolarization-activated cyclic nucleotide-gated channel (HCN3) and causes the up-regulation of HCN3 cell surface expression.^17^^,^^18^ The gene has already been published in the literature as a candidate gene for developmental epileptic encephalopathy. Faqeih et al^17^ reported 7 patients from 4 families with global developmental delay, epilepsy, and variable dysmorphic facial features. The patients were born to consanguineous parents and had biallelic variants in the *KCTD3* gene. Two variants (c.1036_1073del p.(Pro346Thrfs∗4) and c.166C>T p.(Arg56∗)) were described.^17^ The first variant was also described previously in 2 large studies.^19^^,^^20^ In our Biodatabank, we identified 8 additional patients with biallelic *KCTD3* (NM_016121.5) variants in this gene and overlapping neurological phenotype (variants c.1036_1073del p.(Pro346Thrfs∗4), c.528del p.(Arg177fs), c.1547C>G p.(Ser516∗)).

#### *AGR2:* A new GDR - delineation of autosomal recessive RIFTD disease (recurrent respiratory infections and failure to thrive with or without diarrhea) (Limited to Moderate level)

After analyzing exome/genome data and HPO phenotypes, we identified 13 patients from 9 families with a cystic fibrosis (CF)-like phenotype consisting of recurrent lower respiratory infections, failure to thrive and chronic diarrhea, with high morbidity and mortality. All patients had biallelic variants in *AGR2*. We confirmed aberrant *AGR2* transcripts caused by an intronic variant and complete absence of *AGR2* transcripts caused by a large gene deletion, resulting in LoF. Furthermore, transcriptome analysis identified significant downregulation of components of the mucociliary machinery (intraciliary transport and cilium organization), as well as up-regulation of immune processes.^21^ A homozygous variant in this gene has been reported in 2 siblings with severe infantile-onset inflammatory bowel disease and recurrent respiratory infections.^22^ A total of 17 patients have been diagnosed in our laboratory, and 1 prenatal test has been performed. The gene is not (yet) associated to any phenotype in GenCC or HGMD**,** and it has been recently added to OMIM (January 2023); as per our recommendation, (*AGR2* (NM_006408.4) variants: c.182C>T p.(Ala61Val), c.185T>C p.(Leu62Pro), c.211C>A p.(Pro71Thr), c.330+1del p.?, c.349C>T p.(His117Tyr), c.428G>A p.(Gly143Glu), c.456C>G p.(Tyr152∗), and partial gene deletion).

#### *ZNF699*: A new GDR - delineation of autosomal recessive DEGCAGS syndrome (Limited to Moderate level)

We identified and described a new syndrome in 13 patients from 12 families with homozygous LoF variants in this gene. The patients presented with coarse facial features and abnormalities of the cardiovascular, gastrointestinal (gastroesophageal reflux and intestinal atresia), genitourinary (renal dysplasia/hypoplasia and ambiguous genitalia), and skeletal system (syndactyly, preaxial polydactyly, and absent thumbs), and severe neurodevelopmental delay, named as DEGCAGS syndrome (developmental delay with gastrointestinal, cardiovascular, genitourinary, and skeletal abnormalities).^1^ Since then, a total of 21 patients have been reported in our laboratory (*ZNF699* (NM_198535.3) with the variants: c.51_54del p.(Asp17Glufs∗2), c.349dup p.(Ile117Asnfs∗4), c.436_439del p.(Asp146Ilefs∗10), c.1324dup p.(Ser442fs), c.1623_1626del p.(Tyr542Profs∗8), c.994_995del p.(Asp332fs), c.339del p.(Cys113fs), c.217del p.(Gln73fs)). Although little is known about the gene function, the genetic evidence is compelling.

In the period of 1 year, the gene curation efforts led to over 400 patient reports with clinically relevant variants in 61 genes with Moderate, Strong, or Definitive GDR evidence. The complete list of the genes investigated in this study is presented in Supplemental Table 1, together with the level of evidence before and after this study and the relevant references.

## Discussion

Genetic diagnostic processes usually focus on genes with sufficient GDR evidence, which are typically linked to a phenotype in OMIM and/or other databases. Thus, variants in genes with unclear/unknown GDR might remain unreported, leading to missed diagnoses. On the other hand, genes with phenotype associations in OMIM might have insufficient or limited GDR evidence.

In this study, we have implemented the ClinGen clinical validity classification system in a genetic diagnostic setting, aiming to evaluate its feasibility in the clinical practice. Current guidance suggests that only those gene-disease pairs with classifications of Moderate or above should be included in clinical testing.^10^

We investigated 109 GDRs with distinct levels of evidence during a period of 11 months. Using available literature, public databases, and the internal Biodatabank, 61 (56%) GDRs were confirmed with Moderate, Strong, or Definitive evidence, adding these genes to the diagnostic pool of genes (Supplemental Table 1). Although the GDR level of evidence reached may be limited, keeping records of these evaluations paves the way for future GDR discoveries. In some cases, the findings in genes with limited GDR might be reported to the requesting physicians as variants of uncertain significance with the clear label of “research finding” or “gene with limited GDR” and strong recommendation for re-evaluation and follow-up of the specific genetic finding.

Within this study, 59 genes with OMIM phenotype entries were evaluated, and of these, 21 genes (36%) reached evidence points corresponding only to Limited evidence, highlighting the importance of careful GDR evaluation before variant classification and reporting. The disparity in number of genes included in widely used databases illustrates the need of systematic and consistent gene curation (ClinVar 45,461, HGMD 7333, OMIM 6634, and GenCC 4704). Thus, implementation of the ClinGen framework for GDR is not only important to assess newly discovered but also for previously described (putative) gene-phenotype associations.

The process of evidence gathering and evaluation is undoubtfully time consuming, but it should be considered a “must do” step in the diagnostic process, as shown in the current work with 61 new GDR discovered/confirmed. In our experience, the evaluation time can be reduced by having dedicated, highly trained scientists that consistently carry out the process of GDR evaluation. In our team, an average of 3 to 4 hours’ time was deployed for each GDR evaluation, with approximately 10 genes evaluated per month (year 2022). The number of curated genes has doubled in 2023, likely because of the raised awareness of the gene curation process and gained team experience. The cohort of patients referred to our laboratory is highly heterogeneous regarding geographical origin and wide range of genetic diseases that did not preclude the application of the ClinGen framework.

Efforts to systemically annotate gene-disease pair associations, such as the GenCC database, are a major leap toward the curation of such associations. Recently, Riggs et al curated 156 gene-disease pairs, all related to neurodevelopmental disorders and intellectual disability, with 75% being curated as Definitive and 14% as having Limited or Disputed evidence (ClinGen ID/Autism Gene Curation Expert panel).^23^ However, gene discovery outpaces gene annotation as seen in our study with only 8 of 107 evaluated genes having a registered level of evidence in the ClinGen database, 2 of these (*BRSK2* and *PHF21A*) with a Definitive level of evidence. With our study, we provided Moderate or Strong level of evidence for GDR for 11 genes that were not listed in OMIM as having phenotype entity: *FBRSL1*, *AGR2*, *ACBD6*, *C1orf127*, *PAN2*, *VPS50*, *KCTD3*, *NOTCH3* (for autosomal recessive disease), *FAT1*, *NRAP*, and *SCLT1*.

Recently, Clause et al, reported their experience using “reactive” gene curation to support variant classification and reporting of genome sequencing results in a clinical setting.^24^ From 286 GDR evaluated, 86% were classified as having sufficient evidence to support reporting, concluding that reactive GDR curation is a critical step for clinical reporting.^24^ Jauss et al, reported their experience curating 77 genes related to neurodevelopmental disorders for which there was limited public evidence on their clinical significance. The authors did not apply the ClinGen framework but converged on the importance of implementing GDR curation in routine diagnostics.^25^

Furthermore, we should highlight the importance of scientific data and knowledge sharing by using collaborative networks or webtools, such as Matchmaker Exchange^26^ and GeneMatcher (an online portal designed to promote disease gene discovery and data sharing).^27^ Publishing novel GDRs, delineation of new phenotypes, or even case reports supporting previously reported GDRs ensures the detection of additional genes/phenotypes facilitating the diagnosis of more patients. Scientific articles, including confirmed GDRs presented in Supplemental Table 1, have been published or are in the review/preprinting process.^1^^,^^4^^,^^28^

In conclusion, our results demonstrate the importance of careful assessment of gene clinical validity data, along with the use of genetic data repositories. The implementation of ClinGen standardized scoring system provides a method for uniform assessments of the level of GDR evidence. We trust this work will motivate other diagnostic laboratories to implement gene-phenotype curation as a critical step in the process of variant selection, interpretation, and medical reporting.

## List of the links for the data resources used:

OMIM® - https://omim.org/

PubMed - https://pubmed.ncbi.nlm.nih.gov/

ClinGen - https://www.clinicalgenome.org/

GenCC - https://thegencc.org/

Gene2Phenotype - https://www.ebi.ac.uk/gene2phenotype

PanelApp - https://panelapp.genomicsengland.co.uk/

ClinVar - https://www.ncbi.nlm.nih.gov/clinvar/

DECIPHER - https://www.deciphergenomics.org/

Google Scholar - https://scholar.google.com/

Mastermind - https://mastermind.genomenon.com/

GeneReviews - https://www.ncbi.nlm.nih.gov/books/NBK1116/

UniProt - https://www.uniprot.org/

gnomAD - https://gnomad.broadinstitute.org/

HGMD - https://www.hgmd.cf.ac.uk/ac/index.php/

SFARI - https://gene.sfari.org/

SysNDD - https://sysndd.dbmr.unibe.ch/

Geisinger - https://www.geisinger.org/

## Data Availability

The data set that was generated and/or analyzed as part of this study is available from the corresponding author on request.

## Conflict of Interest

Emir Zonic, Mariana Ferreira, Luba M. Pardo, Javier Martini, Maria Eugenia Rocha, Ruxandra Aanicai, Natalia Ordonez-Herrera, Deepa Saravanakumar, Ligia S. Almeida, Inês C. Fernandes, Nishtha Gulati, Sumanth Mannepalli, Ruslan Al-Ali, Catarina Pereira, Omid Paknia, Uros Hladnik, Peter Bauer, Jorge Pinto Basto, and Aida M. Bertoli-Avella are employees of CENTOGENE GmbH. All other authors declare no conflicts of interest.

## Figures and Tables

**Supplementary Figure:**
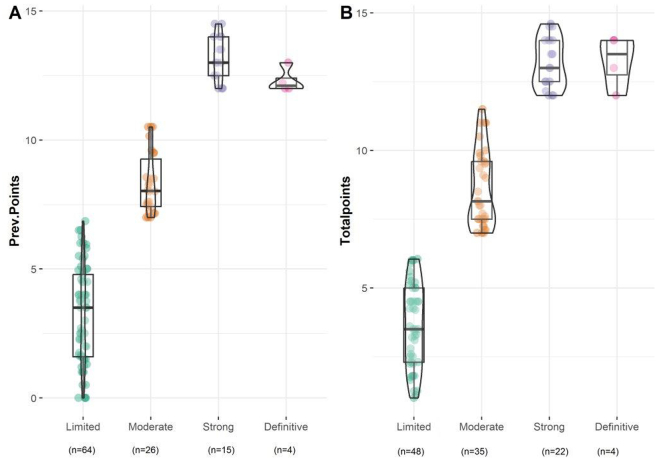
Box plot of the quantitative assessment per category (Limited, Moderate, Strong, and Definitive) before (A) and after (B) considering data from the Biodatabank. An increase in the mean points per category can be seen even when there is no change from category.

## Additional Information

SupplementaryTable1
